# The Effect of Tou Nong San on Transplanted Tumor Growth in Nude Mice

**DOI:** 10.1155/2015/518454

**Published:** 2015-02-18

**Authors:** Liang-Hua Fang, Rui-Ping Wang, Shou-You Hu, Yu-Hao Teng, Wei-Bing Xie

**Affiliations:** ^1^Oncologic Department, Jiangsu Province Hospital of TCM, Nanjing 210029, Jiangsu, China; ^2^Nanjing University of Chinese Medicine, Nanjing 210023, Jiangsu, China

## Abstract

Tou Nong San (TNS) is a traditional Chinese medicinal decoction used to treat sores and carbuncles. It contains four herbal drugs and one animal medicine: Radix Astragaliseu Seu Hedysari, *Angelica sinensis*, Ligustici Chuanxiong, Spina Gleditsiae, and stir-baked Squama Manis. Previous studies have shown that it has anticancer effects. This report validates *in vivo* antitumor properties of TNS. The compounds contained in TNSE were confirmed by liquid chromatographmass spectrometer (LC-MS) analysis. The *in vivo* antitumor activity of TNS extract (TNSE) was tested by feeding it to athymic mice harboring a human colonic tumor subcutaneous xenograft. Toxicity was monitored by recording behavior and weight parameters. Seven compounds were detected in TNSE by LC-MS. TNSE was fed to athymic mice for 2 weeks. No adverse reactions were reported. Compared to the control group, administration of TNSE to tumor bearing mice significantly reduced both tumor weight and volume. The expressions of p-PI3K, p-AKT, p-mTOR, p-p70s6k1, VEGF, and CD31 were significantly reduced, the expression levels of cleaved Caspase-9 and cleaved Caspase-3 were significantly increased in the TNSE groups compared to the control group as determined by western blot and immunohistochemistry. TNSE produced anticolonic cancer effects and the underlying mechanisms involved inhibition of the PI3K/AKT signal transduction pathway, inhibition of angiogenesis, and promotion of apoptotic proteins.

## 1. Introduction

Colorectal cancer (CRC) is one of the leading causes of cancer-related deaths worldwide. In the western world, it ranked third in prevalence and lethality [1]. In China, the incidence of CRC is lower than that in the Western countries, but has significantly increased in recent years with the improvement of current living standards and environmental degradation. Its morbidity has risen year by year [2]. Conventional therapies including surgical resection, chemotherapy, and radiation are often inadequate to treat this disease. Therefore, discovery and development of novel anticancer drugs with more efficacy and/or less toxicity is urgently needed.

Traditional Chinese medicines (TCMs) are widely used as complementary and alternative therapy (CAM) to prevent relapse or metastasis in China. This is especially true for those who failed or finished conventional therapies. However, there is no standard protocol for the use of TCMs on colon cancer.

Tou Nong San (TNS) is derived from Wai Ke Zheng Zong formulated by Chen Shigong. This has been used to treat sores and carbuncles due to weakened body resistance with internal pus and absence of a surface outlet, leading to a failure to expel toxins. It contains five TCMs: Radix Astragaliseu Seu Hedysari 12 g,* Angelica sinensis* 9 g, Ligustici Chuanxiong 9 g, Spina Gleditsiae 4.5 g, and stir-baked Squama Manis 3 g. A previous study has shown that TNSE has effects including antiproliferative action and induction of apoptosis on Raji cells which may be involved in regulation of NF-KB, Bad, Caspase-9, and Caspase-3 [3]. It was also demonstrated that TNSE exhibits a time and dose-dependent preferential cytotoxicity to cultured human colonic cancer cells. However, the* in vivo* efficacy and mechanism of TNSE remained unknown.

This study attempted to decipher the effects of TNSE on colonic cancer xenografts, and provide a rationale for its mechanistic effects.

## 2. Materials and Methods

### 2.1. Reagents

RPMI 1640 medium was purchased from Gibco (CA, USA); fetal calf serum was purchased from Hangzhou Sijiqing Biological Engineering Materials Co., Ltd. (Hangzhou, China). Hematoxylin staining solution was purchased from Nanjing Jiancheng Bioengineering institute. RNase A and antibodies against p-PI3K (Tyr-368), p-AKT (Ser-473), p-mTOR (Ser-2448), p-p70s6k1 (Ser-424), cleaved Caspase-3, cleaved Caspase-9, and *β*-actin were purchased from Nanjing Keygen Biotec Co., Ltd (Nanjing, China). The enhanced chemiluminescence (ECL) kit was purchased from Amersham Life Science (Amersham, UK). Reference compounds calycosin-7-O-*β*-D-glucoside (C1), astragaloside IV (C6), and fenbufen (internal standard, IS) were obtained from the National Institute for the Control of Pharmaceutical and Biological Products (Beijing, China). Formononetin-7-O-*β*-D-glucoside (C2), senkyunolide I (C3), and calycosin (C4) were purchased from Shanghai Dingguo Biotechnology Co., Ltd. (Shanghai, China). Formononetin (C5) was provided by Beijing H&Qitian Chemical Institute (Beijing, China). Z-Ligustilide (C7) was supplied by Nanjing Zelang Chinese Medicine Science and Technology Co., Ltd. (Nanjing, China). The purity of each reference compound was determined to be higher than 96% by normalization of the peak area detected by LC-MS and all reference compounds proved stable in methanol solution over the course of the study. Their structures are shown in Figure 18. HPLC-grade methanol was purchased from Merck (Merck, Darmstadt, Germany). Analytical-grade ethanol was purchased from Nanjing Chemical Reagent Co., Ltd. Deionized water was purified using a Milli-Q system (Millipore, Bedford, MA, USA).

### 2.2. Preparation of Ethanol Extract of TNS

A description of the TNS collections, voucher specimen details, and preparation of ethanol extract of dried leaf powder is listed in an earlier publication [3]. Briefly, ten prescriptions of Tou Nong San, which was composed of washed and dried herbs (375 g), were blended in 3.75 L 80% ethanol (EtOH) (1 : 10 ratio, w/v) for 2 h twice at reflux temperature. After evaporation of organic solvent under reduced pressure, the EtOH extract of Tou Nong San was recovered. This preparation is referred to as TNS extract (TNSE). On average, 66.7 mg residue was obtained per gram of dried herbs. The dry extract was stored in a freezer at −20°C until use.

### 2.3. LC-MS Assay for TNSE

The seven major compounds in TNSE were determined by LC-MS analysis using a Waters 2695 LC system coupled with a Waters ZQ 2000 mass spectrometer. Chromatographic separation was performed on an Agilent Zorbax XDB-C18 (4.6 × 150 mm, 5 *μ*m) column at 25°C. Using water (A) and methanol (B) as the mobile phase, gradient elution was used as follows: 15–75% B from 0–7 minutes with a curve value of 3, 75% B from 7–22 minutes with a curve value of 6, 75–80% B from 22–36 minutes with a curve value of 3, and 80–15% B from 36-37 minutes with a curve value of 6. The re-equilibration time of gradient elution was 6 minutes.

Selected ion response (SIR) mode was employed for mass detection. The target mass ions were set at m/z 469.1 for calycosin-7-O-*β*-D-glucoside, m/z 307.0 for calycosin, m/z 807.4 for astragaloside IV, m/z 191.1 for Z-ligustilide, and m/z 277.3 for fenbufen (IS) in positive mode. Mass ions were simultaneously set at m/z 429.1 for formononetin-7-O-*β*-D-glucoside, m/z 223.1 for senkyunolide I, m/z 267.1 for formononetin, and m/z 253.3 for fenbufen (IS) in negative mode. The TNS EtOH extract was well dissolved in MeOH/H_2_O (v : v = 50 : 50) solution and diluted into an appropriate concentration for LC-MS analysis. All acquisition and analysis of data were controlled by the Mass Lynx 4.1 software.

### 2.4. Cell Line and Cell Culture

Human colon cancer LoVo cells were obtained from Nanjing Keygen Biotech Co., Ltd. (Nanjing, China) and maintained in RPMI 1640 medium, supplemented with 10% fetal calf serum in culture at 37°C in an atmosphere of 5% CO_2_.

### 2.5. Animals

BALB/c mice aged 3-4 weeks with weight of 18–22 g were purchased from SLAC laboratory animal CO. LTD (SCXK-2007-004, Shanghai, China) and were housed five per cage in a specific pathogen-free (SPF) environment. The animals were allowed to acclimate to the housing facilities for 7 days before the experiments began. Animal handling procedures were carried out in accordance with the P. R. China legislation on the use and care of laboratory animals and approved by Experimental Animal Ethical Committee of Jiangsu Province Hospital of Traditional Chinese Medicine (Nanjing, China).

### 2.6. Xenograft Assay and Treatment

The LoVo cell suspension was subcutaneously injected (1 × 10^7^/mL in 200 *μ*L PBS) into the right flank of the BALB/c mice. The tumor volume (*V*) was calculated using the following equation: *V* = 1/2 × *a* × *b*2, where *a* and *b* are the longest and the shortest diameters of the tumor mass (in millimeters), respectively. When the tumors reached approximately 80 mm^3^, the animals were then divided randomly into the following four groups of eight animals each: Group A received normal saline (NS), Group B–D; 4 g, 2 g and 1 g/kg TNSE respectively via oral administration. Each animal received a total of 14 doses.

Toxicity profile in the mice fed with the listed concentrations of TNSE was recorded. Qualitative physical features as indicated by red eye, diarrhea, and lethargy were observed daily. The function of liver and renal also was detected at the end of the experiment. Quantitative changes in body weight and tumor volume were measured every two days by a single individual using balance and Vernier caliper. The tumor volume (*V*) was calculated using the formula: *V* = 1/2 × *a* × *b*2. Tumor bearing mice were euthanized 28 days after tumor cell injection. They were treated for 14 days during which time each set of animals received 14 oral doses of the drug or NS. At the end of the study, the tumor was weighed and volume measured using a caliper. The tumor inhibitory rate was calculated using the following formula: tumor inhibition ratio (%) = [(*C* − *T*)/*C*] × 100%, where *C* is the tumor weight average of the blank control (normal saline), and *T* is that of the treated group. Tumor tissues were harvested for tissue processing. One portion of each tumor underwent tumor histology observation by HE staining, another went to detection of proteins by immunohistochemistry, and the remaining portions of the tissues were frozen in liquid nitrogen for western blot analysis.

### 2.7. Immunohistochemistry

The expression of p-PI3K, p-AKT, p-mTOR, p-p70s6k1, cleaved Caspase-3, cleaved Caspase-9, VEGF and CD31 in xenografts was detected by Streptavidin/Peroxidase (SP) immunohistochemistry according to the kit instructions. The negative control was performed with PBS instead of primary antibody.

Paraffin embedded tissue sections were deparaffinized by xylene, sequentially rehydrated by 100% ethanol, 75% ethanol, 50% ethanol, and then distilled water. The antigen retrieval step was accomplished by heating the slides in citrate buffer (Target Retrieval Solution, Citrate pH 6, Keygentec, China), buffer (Target Retrieval Solution, Citrate pH 6, Keygentec, China), and protein block (Background Sniper, Biocare-Medical, USA) for 10 minutes each.

The slides were incubated with primary antibody (Cell Signaling Tech., USA) for 30 minutes, secondary antibody (4 plus Biotinylated Universal Goat Link; Biocare-Medical, USA) for 15 minutes, and finally Streptavidin-HRP 4 plus Streptavidin HRP Label and (DAB) (Betazoid DAB Chromogen Kit; Biocare-Medical, USA) for 15 minutes. 3,3′ Diaminobenzidine (DAB) (Betazoid DAB Chromogen Kit; Biocare-Medical, USA) was used to stain the antibody positive regions brown.

The immunoreactivity was evaluated using an immunoreactivity score (IRS), which takes into account both the percentage of positive cells and staining intensity. This scoring method avoids the disadvantages of scoring single positive cells or positive intensity scoring and more accurately reflects the results of immunohistochemical reactions.

### 2.8. Western Blot Analysis

To evaluate p-PI3K, p-AKT, p-mTOR, p-p70s6k1, cleaved Caspase-3, cleaved Caspase-9, VEGF, and CD31 protein, 0.2 g of tissue was removed from liquid nitrogen and washed three times with precooled PBS. The tissue was then ground into small pieces. The tissue was stirred in 10 volumes of lysis buffer and centrifuged at 4°C for 10 minutes. The total protein was then isolated. Concentration was determined using the Bradford method. The proteins were separated by electrophoresis with sodium dodecyl sulfate-polyacrylamide gel (SDS-PAGE) and then transferred onto polyvinylidene difluoride (PVDF) membranes. After blocking with 5% nonfat dry milk in TBST (20 mM Tris-HCl, 150 mM NaCl, and 0.05% Tween-20) for 1 h at room temperature, the membranes were incubated with primary antibodies overnight at 4°C. The membranes were then incubated with HRP conjugated goat anti-rabbit secondary antibodies for 2 h at room temperature. The membranes were washed three times with TBST for 10 minutes. The specific p-PI3K, p-AKT, p-mTOR, p-p70s6k1, cleaved Caspase-3, cleaved Caspase-9, VEGF, and CD31 bands were developed using ECL reagent and imaged using a gel scanner. The protein levels were normalized to GAPDH as a reference.

### 2.9. Statistical Analysis

All results were analyzed with SPSS 10.0 software. The data were expressed as means ± standard deviation. The paired *t*-test and *χ* ^2^ test were used for statistical analysis between groups. The correlations were evaluated by using the Pearson correlation coefficient. *P* value less than 0.05 was considered to be statistically significant.

## 3. Results

### 3.1. Composition of TNSE

**Figure 1:**
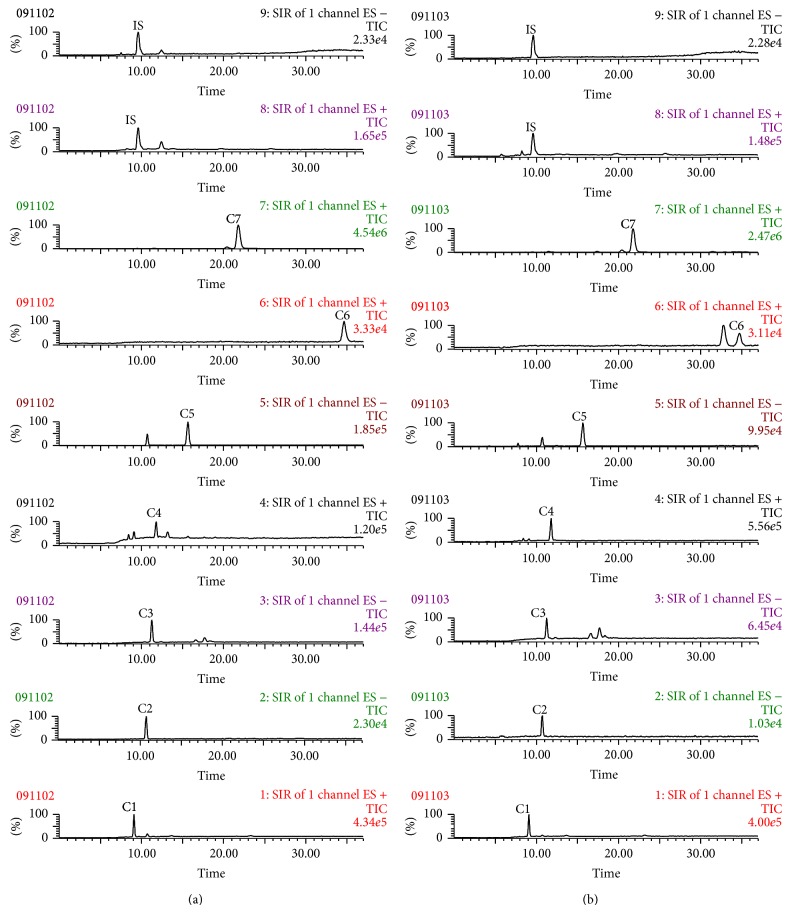
Representative SIR chromatograms of seven reference standards and IS from the LC-MS analysis of the mixture of reference compounds. (a) TNSE (b) C1, calycosin-7-O-*β*-D-glucoside; C2, formononetin-7-O-*β*-D-glucoside; C3, senkyunolide I; C4, calycosin; C5, formononetin; C6, astragaloside IV; C7, Z-ligustilide; IS, fenbufen.

The constituents of TNSE were identified by comparing the SIR chromatograms of the TNSE with that of the mixture of reference compounds. As shown in Figure 1, the presence of seven major constituents in the extract was confirmed by the common retention time in their corresponding ion channels of both the extract and the compound mixture. After being fully validated with respect to linearity, precision, repeatability, and accuracy, the established LC-MS method was then applied to quantify the seven constituents in the EtOH extract of TNS for the standardization of the TNSE. The composition of the TNSE used for the experiments is shown in Table 1.

### 3.2. Effect of TNSE on Xenograft Growth in Nude Mice

**Figure 2:**
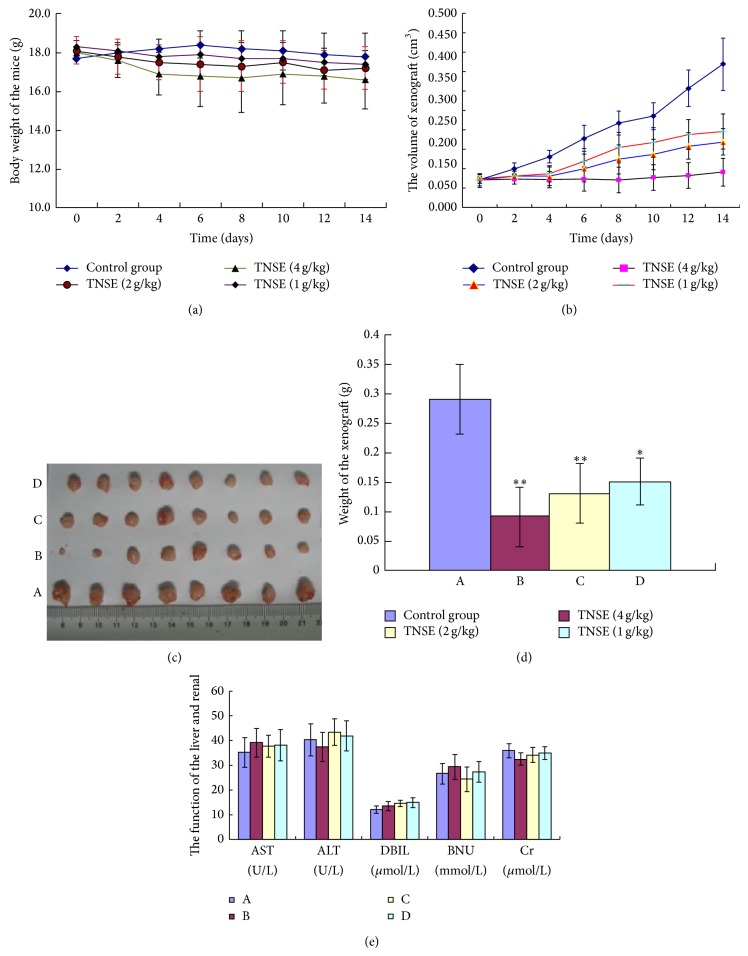
The effects of different dose of TNSE on the growth of LoVo cells-xenografts in nude mice. (a) The tumor volumes were recorded at 2-day intervals. TNSE inhibited the growth of the transplanted tumors, and the volume of the tumors in the TNSE treated group was much smaller than that in the control group (*P* < 0.05). Data are represented as means ± SD (*n* = 8). (b) Body weight was evaluated at 2-day intervals in all groups. In the control group, the body weights of mice were higher than that in the TNSE treated groups, but there have been no significance among these four groups (*P* > 0.05). Data are represented as means ± SD (*n* = 8). (c) The xenografts in the four groups at the conclusion of the experiment. A: control (NS), B: TNSE (4 g/kg), C: TNSE (2 g/kg), and D: TNSE (1 g/kg). (d) TNSE could inhibit the growth of the transplanted tumor. With increasing dose, the weight of xenografts decreases. (e) The function of liver and renal. A: control (NS), B: TNSE (4 g/kg), C: TNSE (2 g/kg), and D: TNSE (1 g/kg). Data are represented as means ± SD (*n* = 8). ^*^ *P* < 0.05 versus Control group; ^**^ *P* < 0.01 versus Control group.

For the duration of treatment (14 days) none of the mice exhibited any signs of physical discomfort. Quantitatively, all mice lost 5–10% weight, which is likely attributable to the cancer cells. At the start of treatment (day 0), mean body weight was 23.5 g in control and treatment groups. At the end of treatment (day 14) mean body weight was 21.2 g in all groups. Though not significant, (*P* > 0.67) untreated mice lost more weight (Figure 2(a)). Furthermore, the function of liver and renal was normal in these four groups (Figure 2(e)), which indicated that TNSE was safe for the mice and the toxicity of TNSE was very little.

### 3.3. Antitumor Activity

TNSE significantly decreased the growth of human colonic LoVo cancer cells transplanted subcutaneously in athymic mice. Responses to the treatment regimen were sequentially monitored by measuring tumor size. Tumors were smaller in the treatment groups, and gradually decreased in size with increased dose. The initial volume of the xenograft was approximately 0.07 cm^3^. At the conclusion of the study, the volume of the mass in the control group was 0.369 ± 0.067 cm^3^, but 0.091 ± 0.045 cm^3^ in the high dose TNSE group (Figure 2(b)). The resected tumor mass of the TNSE treated group was significantly smaller (mean 0.09 ± 0.046 g versus 0.29 ± 0.06 g, *P* < 0.01) (Figures 2(c) and 2(d)).

### 3.4. TNSE Mediated Differential Regulation of Genes Involved in Cell Proliferation, Apoptosis, and Angiogenesis

Further exploration was done concerning whether inhibition of the PI3K/AKT pathway was involved in TNSE treated groups with IHC, as it plays a critical role in proliferation, resistance to apoptosis, angiogenesis, and metastasis in colorectal cancers.

**Figure 3:**
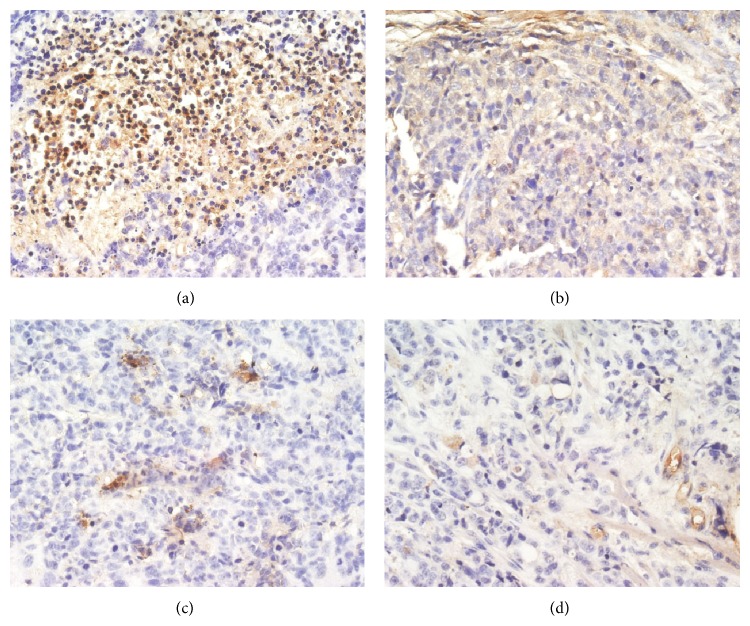
Expression of p-PI3K protein in tumor tissues from control and treated mice (400×). TNSE significantly decreased the expression of p-PI3K (*P* < 0.001). (a) NS group; (b) 1 g/kg TNSE group; (c) 2 g/kg TNSE group; (d) 4 g/kg TNSE group.

**Figure 4:**
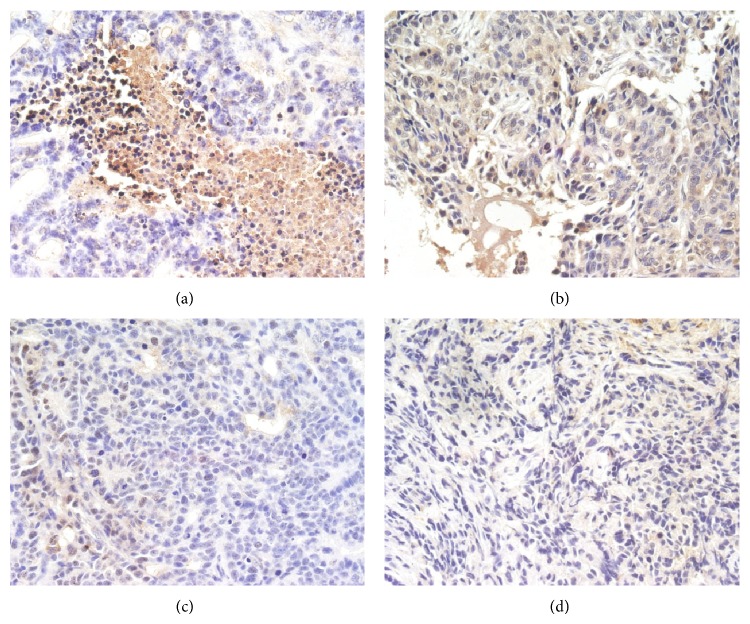
Expression of p-AKT protein in tumor tissues from control and treated mice (400×). TNSE significantly decreased the expression of p-AKT (*P* < 0.001). (a) NS group; (b) 1 g/kg TNSE group; (c) 2 g/kg TNSE group; (d) 4 g/kg TNSE group.

**Figure 5:**
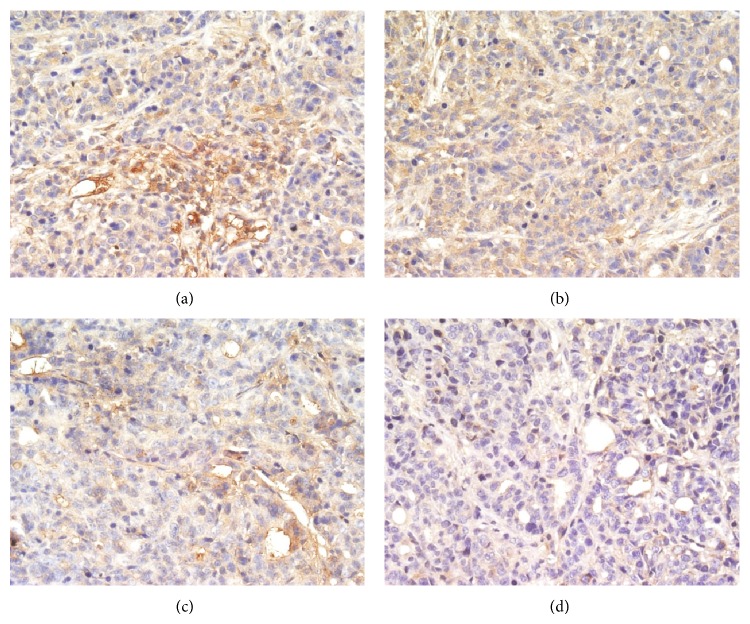
Expression of p-mTOR protein in tumor tissues from control and treated mice (400×). TNSE significantly decreased the expression of p-mTOR (*P* < 0.001). (a) NS group; (b) 1 g/kg TNSE group; (c) 2 g/kg TNSE group; (d) 4 g/kg TNSE group.

**Figure 6:**
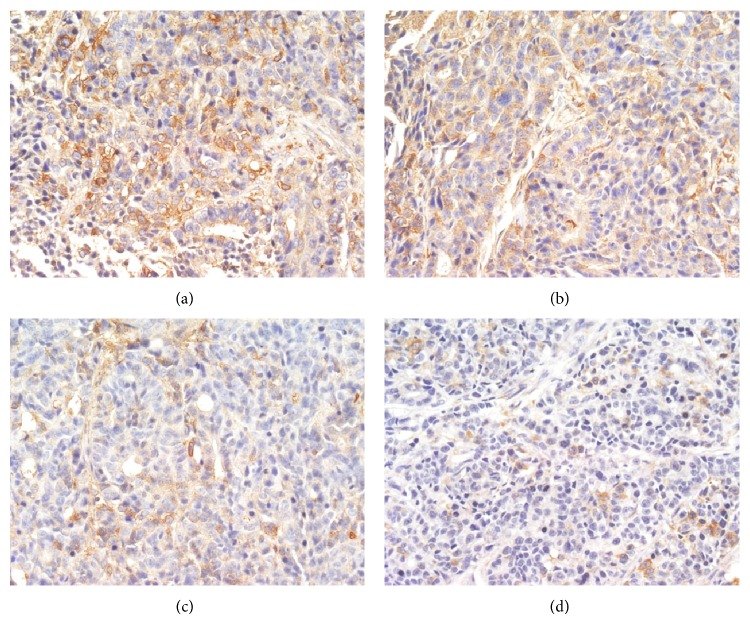
Expression of p-p70^s6k1^ protein in tumor tissues from control and treated mice (400×). TNSE significantly decreased the expression of p-p70^s6k1^ (*P* < 0.001). (a) NS group; (b) 1 g/kg TNSE group; (c) 2 g/kg TNSE group; (d) 4 g/kg TNSE group.

**Figure 7:**
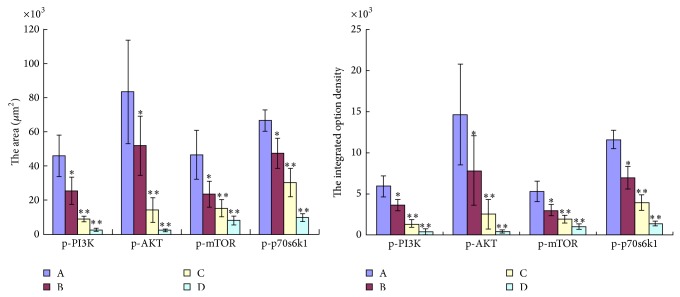
The positive area of p-PI3K, p-AKT, p-mTOR, and p-p70^s6k1^ and integrated option density (IOD) were determined. A: NS group; B: 1 g/kg TNSE group; C: 2 g/kg TNSE group; D: 4 g/kg TNSE group. Values given are the means ± SD for 8 tumor specimens in each group.

**Figure 8:**
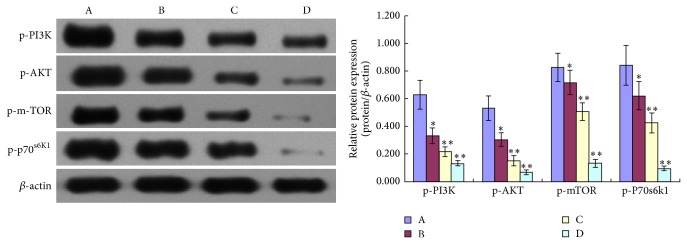
Expression of p-PI3K, p-AKT, p-m-TOR, and p-p70^s6k1^ proteins in xenograft. The effects of the treatment with TNSE on protein expression were measured in multiple samples (*n* = 8) of xenografts by Western blot. Densitometric analysis was evaluated by Syngene G:BOX Chemi XR5 (English) and results were expressed as relative units. A: NS group; B: 1 g/kg TNSE group; C: 2 g/kg TNSE group; D: 4 g/kg TNSE group. Values given are the means ± SD for 8 tumor specimens in each group. ^*^ *P* < 0.05, ^**^ *P* < 0.01, when compared to control group.

When probed with phosphospecific PI3K antibody, there was a notable decrease in staining intensity for phosphorylated PI3K in tumors from TNSE treated mice (Figures 3(b)–3(d)). Tumors from control mice tested strongly positive for the presence of p-PI3K (Figure 3(a)). Interestingly, the immune staining of the downstream activated molecules of PI3K, including p-AKT, p-m-TOR, and p-p70^s6k1^ was also more predominantly observed in the xenografts from control mice (Figures 4(a), 5(a), 6(a), and 7) and staining intensities gradually grew weaker in the TNSE treated groups with increasing dose with it being sparsely observed in the high dose TNSE group (Figures 3(d), 4(d), 5(d), 6(d), and 7). This indicates TNSE prevented phosphorylation of PI3K, Akt, m-TOR, and p70^s6k1^ in human colonic tumor grown subcutaneously in athymic mice. The Western Blot results are in agreement with IHC. In brief, the level of p-PI3K, p-Akt, p-mTOR, and p-p-70^s6k1^ proteins in transplanted tumors in the TNSE groups were lower than those of control group (*P* < 0.05) (Figure 8) and the downregulation was more predominant in higher dose groups (*P* < 0.05). These results validated TNSE as an inhibitor of the activation of the PI3K/AKT pathway in LoVo cell transplanted tumors, which might be responsible for the inhibition of the xenograft growth.

**Figure 9:**
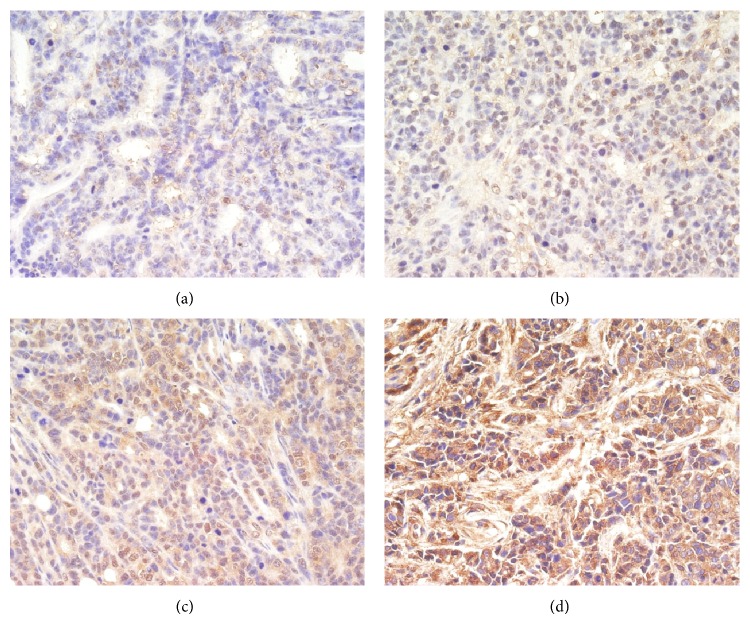
The expression of Caspase-3 in the transplanted tumors (400×). TNSE significantly decreased the expression of Caspase-3 (*P* < 0.001). (a) NS group; (b) 1 g/kg TNSE group; (c) 2 g/kg TNSE group; (d) 4 g/kg TNSE group.

**Figure 10:**
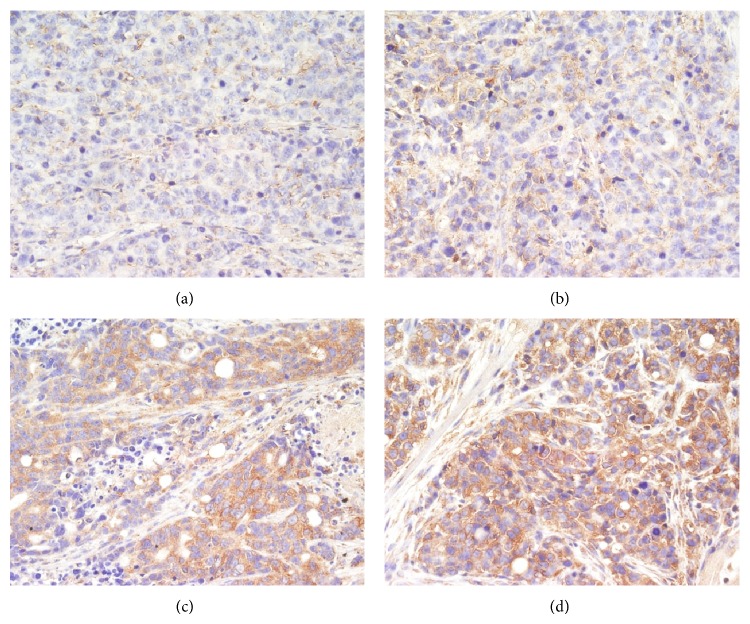
The expression of Caspase-9 in the transplanted tumors (400×). TNSE significantly decreased the expression of Caspase-9 (*P* < 0.001). (a) NS group; (b) 1 g/kg TNSE group; (c) 2 g/kg TNSE group; (d) 4 g/kg TNSE group.

**Figure 11:**
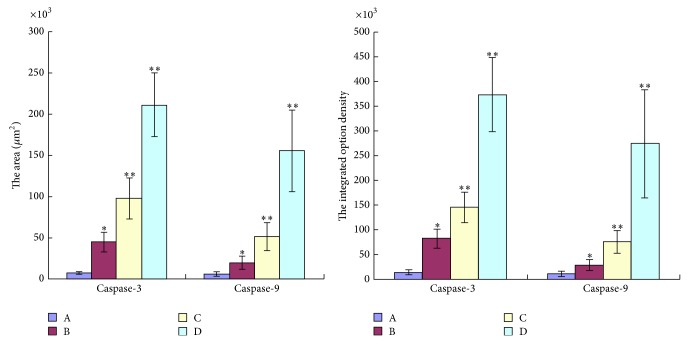
The positive area of Caspase-3 and Caspase-9 with integrated option density determined. TNSE significantly decreased the expression of Caspase-3 and Caspase-9. A: NS group; B: 1 g/kg TNSE group; C: 2 g/kg TNSE group; D: 4 g/kg TNSE group. Values given are the means ± SD for 8 tumor specimens in each group.

**Figure 12:**
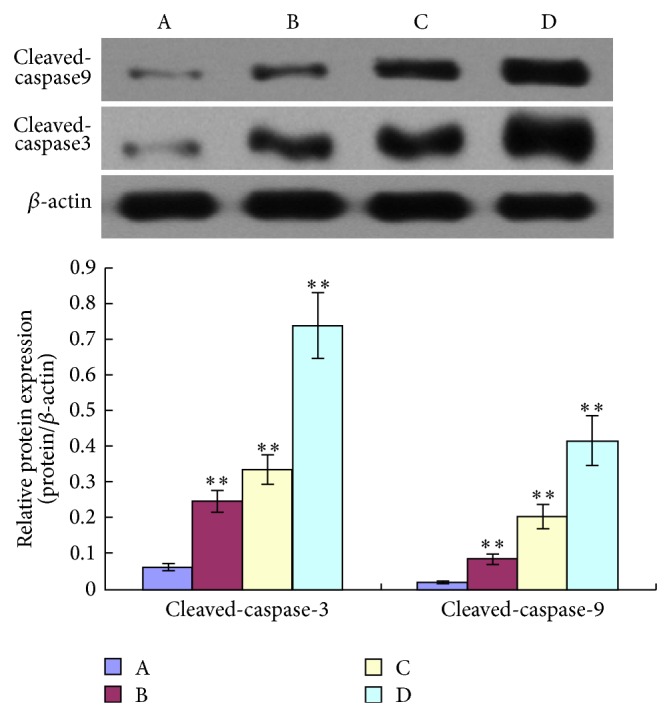
Expression of cleaved Caspase-3 and cleaved Caspase-9 proteins in xenograft. A: NS group; B: 1 g/kg TNSE group; C: 2 g/kg TNSE group; D: 4 g/kg TNSE group. Values given are the means ± SD for 8 tumor specimens in each group. ^*^ *P* < 0.05, ^**^ *P* < 0.01, when compared to control group.

Immunohistochemistry (IHC) for cleaved Caspase-3 and cleaved Caspase-9 produced positively stained tumors in TNSE treated mice, with stain intensity growing with increased dose (Figures 9(b)–9(d), 10(b)–10(d), and 11). Untreated controls, however, sparsely stain positive for activated Caspase-3 and Caspase-9 (Figures 9(a), 10(a) and 11). Western Blot further confirmed the activation of Caspase-3 and Caspase-9 in TNSE treated groups (Figure 12). Increased staining intensities indicated the increased levels of target proteins induced by TNSE. The above two results confirmed TNSE treated tumors were undergoing apoptosis.

**Figure 13:**
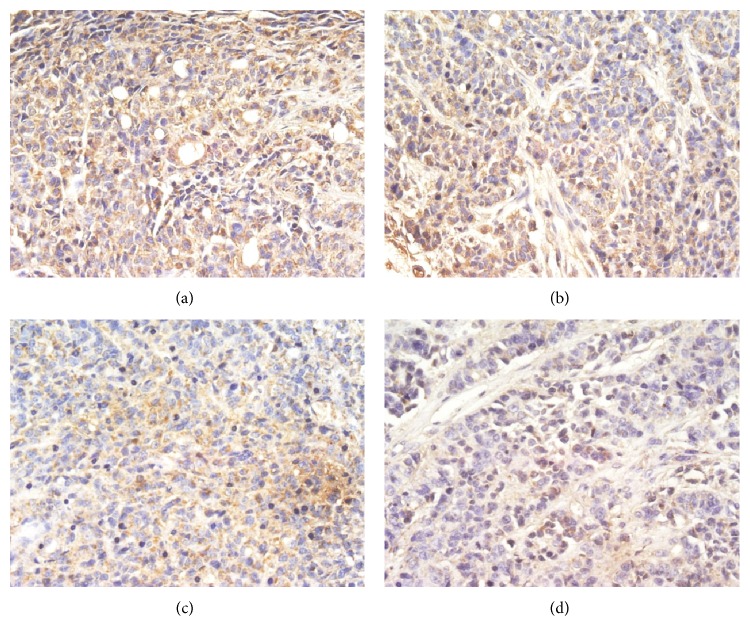
The expression of VEGF in the transplanted tumors (400×). (a) NS group; (b) 1 g/kg TNSE group; (c) 2 g/kg TNSE group; (d) 4 g/kg TNSE group. Values given are the means ± SD for 8 tumor specimens in each group.

**Figure 14:**
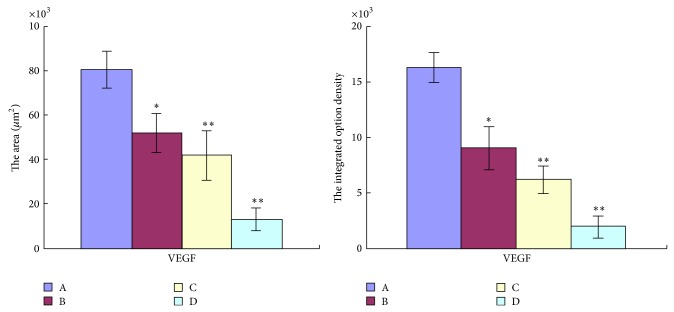
The positive area of VEGF and integrated option density of it were determind. A: NS group; B: 1 g/kg TNSE group; C: 2 g/kg TNSE group; D: 4 g/kg TNSE group. Values given are the means ± SD for 8 tumor specimens in each group.

**Figure 15:**
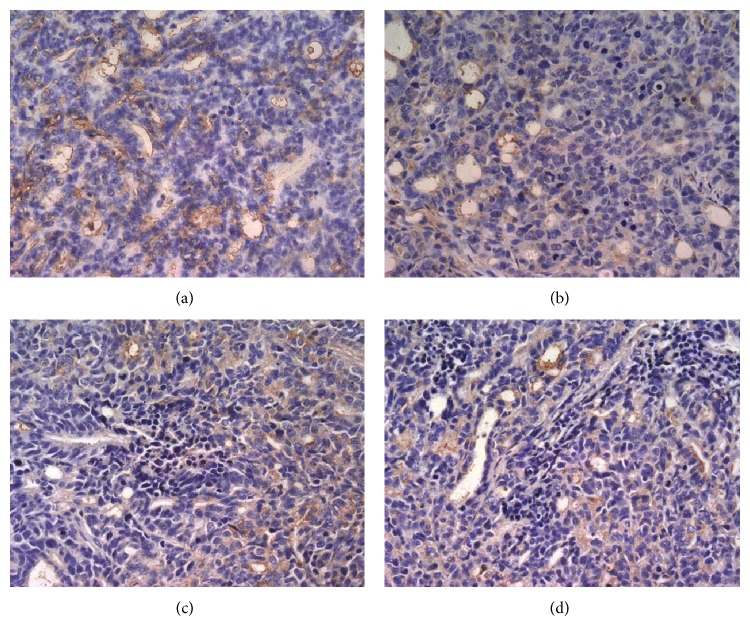
The expression of CD31 in the transplanted tumors (400×). Densitometric analysis was evaluated by Image-Pro Plus 6.0 (Media Cybernetics, American) which automatically calculates the amount of microvasculature. (a) NS group; (b) 1 g/kg TNSE group; (c) 2 g/kg TNSE group; (d) 4 g/kg TNSE group. Values given are the means ± SD for 8 tumor specimens in each group.

**Figure 16:**
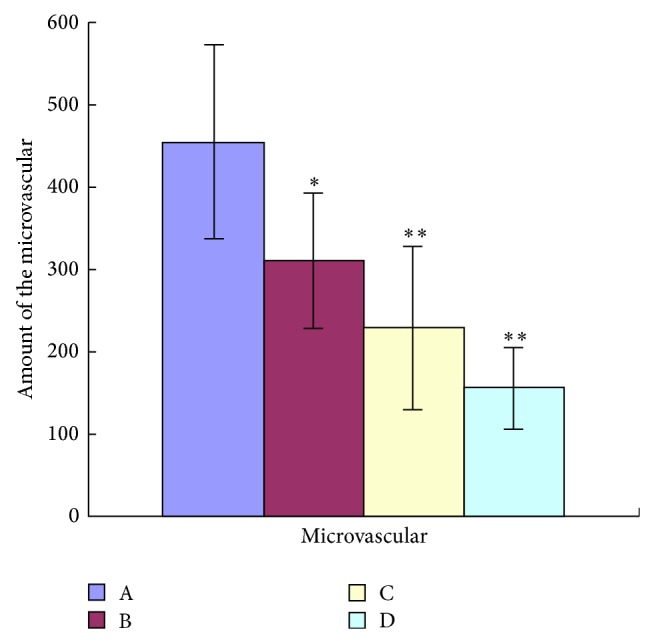
The amount of the microvasculature gradually decreased with increasing TNSE dose. Densitometric analysis was evaluated by Image-Pro Plus 6.0 (Media Cybernetics American). A: NS group; B: 1 g/kg TNSE group; C: 2 g/kg TNSE group; D: 4 g/kg TNSE group. Values given are the means ± SD for 8 tumor specimens in each group.

To investigate the effects of TNSE on tumor angiopoiesis, VEGF and CD31 proteins were evaluated by IHC and Western Blot. The control group had the highest amount of microvasculature and positive staining of VEGF and CD31 was predominantly observed in the cytoplasm of tumor cells (Figures 13(a), 14, 15(a) and 16). The staining intensity gradually weakened with increased TNSE dose. In the high dose TNSE group, VEGF and CD31 staining was sparsely observed and the least microvasculature was observed within the xenograft (Figures 13(d), 14, 15(d) and 16).

**Figure 17:**
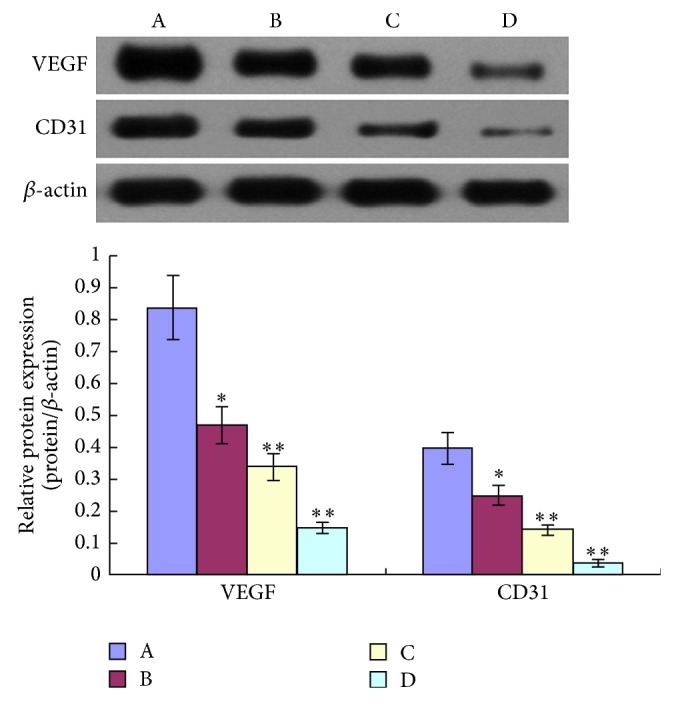
Expression of VEGF, CD31 proteins in xenograft. A: NS group; B: 1 g/kg TNSE group; C: 2 g/kg TNSE group; D: 4 g/kg TNSE group. Values given are the means ± SD for 8 tumor specimens in each group. ^*^ *P* < 0.05, ^**^ *P* < 0.01, when compared to control group.

**Figure 18:**
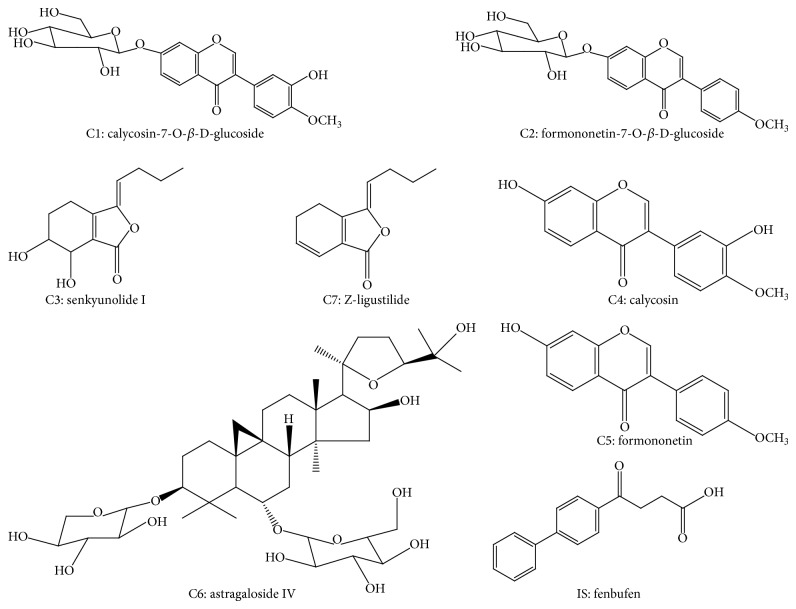
The chemical structures of 7 selected marker compounds and IS.

Western Blot for VEGF and CD31 expressed highly in tumors obtained from untreated controls and expression decreased by degree with increased TNSE dose (*P* < 0.05) (Figure 17). This agrees with the results from IHC. Together, these two results suggest that TNSE could inhibit the neovascularization in tumors, which is important in tumorigenesis and tumor progression.

## 4. Discussion

The purpose of this study was to investigate,* in vivo*, whether TNSE had anticancer properties and its potential mechanism(s). Previous studies have found that TNSE has anticancer effects* in vitro*, including the inhibition of proliferation and induction of apoptosis on human lymphoma cell line Raji cells [3]. This encouraged further research. Presence of antitumor activity and absence of toxicity in the animal studies may substantiate the investigation of TNSE as a cancer therapy. Initially the LC-MS was applied to analyze the components of the TNSE.

Water extract was the traditional preparation for TNS in clinical application; however, in previous studies (this paper has been accepted), the ethanol extract of TCMs usually contained more marker compounds and hence exhibited relatively stronger potency. Therefore, in our current study, the ethanol extraction of TNS was investigated. Seven major components (Figure 1, Table 1) in TNSE were identified, including calycosin-7-O-*β*-D-glucoside, formononetin-7-O-*β*-D-glucoside, senkyunolide I, calycosin, formononetin, astragaloside IV, and Z-ligustilide. Z-Ligustilide appears to be the major component of TNSE, with a concentration of 5464.5 ug/mL. Several studies identified its anticancer effects [4, 5]. Other active pharmacological constituents had been confirmed to possess antitumor effects [4, 6–8]. This established a pharmacological basis for TNSE anticancer effects. Though TNSE is comprised of a myriad of compounds, it is believed that the compounds in TNSE work in synergy with each other to produce a more pronounced effect.

In the present study, the anticancer effects of the TNSE on mice bearing LoVo cells were demonstrated. As shown in Figure 2, the weight and volume of the xenograft was gradually decreased with increased TNSE dose (*P* < 0.01). There was no difference in the body weight of mice among the four groups (*P* > 0.05) which confirmed that TNSE is a prescription with excellent anticancer effect against colonic cancer and little toxicity.

Next, an immunohistochemical staining was performed of four molecules in the PI3K/Akt/mTOR signaling axis including PI3K, Akt, mTOR, and p-70^s6k1^ within the primary tumor. This pathway plays a critical role in the proliferation, resistance to apoptosis, angiogenesis, and metastasis that is central to the development and maintenance of colorectal cancers [9]. PI3K is activated upon growth factors binding to their receptors. Activated PI3K leads to the activation of Akt by phosphorylation at Ser473 and Thr308 [10]. Akt activates several downstream targets including mTOR. Deregulation of mTOR signaling occurs in several human tumors including colon cancer [9]. Following that, mTOR associates with Raptor (mTORC1 complex) to phosphorylate p70^S6K1^, leading to increased cell proliferation [11]. It has been reported that the PI3K/AKT/mTOR pathway components p85*α*, AKT1, AKT2, p-mTOR, and p-p70^S6K1^ are highly overexpressed in CRC, which may contribute to the growth and progression of CRC [12]. This study's observations are consistent with these published reports [12]. In the control group, these four molecules within the primary tumor were highly expressed. Positive PI3K, Akt, mTOR, and p70^S6K1^ immunostaining was predominantly observed in the cytoplasm or on the plasmalemma of tumor cells (Figures 3(a), 4(a), 5(a) and 6(a)). Their positively expressed areas, which were analyzed by Image-Pro Plus 6.0 system (Media Cybernetics American), were much larger than those in TNSE treated groups (Figure 7, *P* < 0.05 or *P* < 0.01). Meanwhile, their integrated option density (IOD) was obviously higher than those in the TNSE treated groups (Figure 7, *P* < 0.05 or *P* < 0.01). The expression of these four proteins in the tumor tissues obtained from control and TNSE treated mice by Western Blot was also examined. Treatment with TNSE resulted in significantly lower levels of phosphorylation of Akt, mTOR, and p70^S6K1^ than those in the control group (Figure 8, *P* < 0.05 or *P* < 0.01). Further confirmation of the down regulation was obtained by immunohistochemistry for the proteins in the xenograft tissues. These data suggest that TNSE significantly affects expression of PI3K/Akt/mTOR signaling–related proteins, which might contribute to the inhibitory effects of TNSE on the xenograft tumors.

Additionally, AKT can concurrently interact with and inhibit the initiator apoptotic enzyme, Caspase-9 [13], which is the apical Caspase in the intrinsic or mitochondria-initiated apoptosis pathway [14]. Once activated, it cleaves and activates effector caspases such as Caspase-3 and Caspase-7 which in turn cleave numerous cellular substrates, resulting in cell death [15]. Most cancer cells can block apoptosis, which allows them to survive. Therefore, the induction of apoptotic cell death is an important mechanism in many anticancer drugs [16–18].

In the present study, the IHC and Western Blot results both showed that the active proteins cleaved Caspase-3 and cleaved Caspase-9 were markedly upregulated in the transplanted tumor of the TNSE treated groups compared with the control group (*P* < 0.01, *P* < 0.05, Figures 9(b)–9(d), 10(b)–10(d), 11, and 12), which suggests that TNSE could induce tumor apoptosis and might contribute to the decrease of the tumor size. Accompanied by marked decreased expression of p-AKT, it also suggests that the apoptosis induction of TNSE might relate to regulation of the PI3K/AKT/Caspase-9, Caspase-3 pathway.

Besides activation of Caspase-3 and Caspase-9, it is interesting to note recorded decreases in VEGF protein in TNSE treated tumors. A previous study showed that VEGF is unique among angiogenic factors by virtue of its paracrine effect on the proliferation and motogenesis of endothelial cells [19]. The pattern of VEGF expression suggests its involvement in the development and maintenance of the normal vascular system and in tumor angiogenesis [19]. Angiogenesis is an essential step in tumor growth and metastasis [20]. In colon cancer, VEGF and its receptors are overexpressed which plays an important role in angiogenesis and promotion of tumor growth [21, 22].

The study results showed that VEGF was overexpressed in tumor tissue of the control group (Figures 13(a), 14, and 17), which was in agreement with two published studies [21, 22]. The expressions of VEGF were significantly downregulated after treatment with TNSE (Figures 13(b)–13(d), 14, and 17). Furthermore, the inhibitory effect was more pronounced with increasing dose. Accompanied by the high expression of VEGF in the xenograft in the control group, the microvessel density (MVD), which was evaluated by the expression of CD31 protein, was also high in the control group (Figures 15(a) and 16). CD31 is also related to neovascularization and widespread distribution throughout the entire vascular tree [23, 24]. After treatment with TNSE, the MVD was notably decreased (Figures 15(b)–15(d) and 16). These results indicate that TNSE is a potential antiangiogenic agent.

In light of the present data and the context of previous findings, it is proposed that TNSE could inhibit the activation of PI3K/AKT/mTOR signal pathway and activate Caspase-3 and Caspase-9, as well as inhibiting the angiogenesis. These events may be responsible for the inhibition of the growth of the xenograft tumors in treatment groups. Notably, these effects were more pronounced in higher dose groups. These results are encouraging and merit further investigation. Understanding the molecular mechanism of action of TNSE on tumor specific markers is fundamental to establish the mechanism of the antitumor properties of TNSE.

## 5. Conclusion

This study demonstrates for the first time that Tou Nong San has excellent* in vivo* anticancer properties which may be closely associated with the regulation of PI3K/Akt/mTOR pathway, activation of apoptosis, and inhibition of angiogenesis. It may be evidence to support the clinical benefit to CRC patients and substantiates the investigation of TNSE as an anticancer drug in humans.

## Figures and Tables

**Table 1:** Characterization of marker compounds of TNSE.

Assigned identity	Content in TNSE (ug/mL)
Calycosin-7-O-b-D-glucoside	76.9
Formononetin-7-O-b-D-glucoside	41.5
Senkyunolide I	385.5
Calycosin	60.9
Formononetin	38.7
Astragaloside IV	21.9
Z-Ligustilide	5464.5
